# Ecological Niche of the 2003 West Nile Virus Epidemic in the Northern Great Plains of the United States

**DOI:** 10.1371/journal.pone.0003744

**Published:** 2008-12-05

**Authors:** Michael C. Wimberly, Michael B. Hildreth, Stephen P. Boyte, Erik Lindquist, Lon Kightlinger

**Affiliations:** 1 Geographic Information Science Center of Excellence, South Dakota State University, Brookings, South Dakota, United States of America; 2 Department of Biology, South Dakota State University, Brookings, South Dakota, United States of America; 3 South Dakota Department of Health, Pierre, South Dakota, United States of America; University of Liverpool, United Kingdom

## Abstract

**Background:**

The incidence of West Nile virus (WNv) has remained high in the northern Great Plains compared to the rest of the United States. However, the reasons for the sustained high risk of WNv transmission in this region have not been determined. To assess the environmental drivers of WNv in the northern Great Plains, we analyzed the county-level spatial pattern of human cases during the 2003 epidemic across a seven-state region.

**Methodology/Principal Findings:**

County-level data on WNv cases were examined using spatial cluster analysis, and were used to fit statistical models with weather, climate, and land use variables as predictors. In 2003 there was a single large cluster of elevated WNv risk encompassing North Dakota, South Dakota, and Nebraska along with portions of eastern Montana and Wyoming. The relative risk of WNv remained high within the boundaries of this cluster from 2004–2007. WNv incidence during the 2003 epidemic was found to have a stronger relationship with long-term climate patterns than with annual weather in either 2002 or 2003. WNv incidence increased with mean May–July temperature and had a unimodal relationship with total May–July precipitation. WNv incidence also increased with the percentage of irrigated cropland and with the percentage of the human population living in rural areas.

**Conclusions/Significance:**

The spatial pattern of WNv cases during the 2003 epidemic in the northern Great Plains was associated with both climatic gradients and land use patterns. These results were interpreted as evidence that environmental conditions across much of the northern Great Plains create a favorable ecological niche for Culex tarsalis, a particularly efficient vector of WNv. Further research is needed to determine the proximal causes of sustained WNv transmission and to enhance strategies for disease prevention.

## Introduction

West Nile virus (WNv) is indigenous to Africa, Asia, Europe, and Australia, and was first identified in North America in the New York City metropolitan area during the summer of 1999 [1]. In 2002 widespread WNv cases were reported in the Midwest and south-central states. In 2003 the focus of the West Nile epidemic shifted westward to the Great Plains and the Front Range of the Rocky Mountains. WNv incidence rates, expressed in cases per 100,000 people, were 97 in North Dakota, 135 in South Dakota, and 112 in Nebraska during the 2003 epidemic. In subsequent years, WNv incidence in the northern Great Plains has been lower than in 2003 but has remained substantially higher than in other parts of the United States [2]. However, we still have only a limited understanding of the environmental factors that are responsible for the elevated risk of WNv in this region. To improve our knowledge of the environmental determinants of WNv, we analyzed the influences of climate, weather, land cover, and land use on the spatial pattern of the 2003 epidemic in the northern Great Plains.

Temperature and precipitation are both important environmental drivers of WNv amplification. Increased temperature results in more rapid maturation of mosquitoes and decreases the extrinsic incubation period of the virus [3]. Temperature variability in space and time influences the relative abundances of different mosquito species [4], and temperature-driven shifts in mosquito feeding behavior may trigger a shift from WNv amplification within avian communities to human transmission [5], [6]. A study of WNv in wild bird communities in Georgia found that seroprevalence was highest in the warmest portions of the state [7]. Similarly, the incidence of WNv in Alberta was higher in the relatively warm grassland ecoregion compared with the cooler boreal and mountain ecoregions [8], and the incidence of WNv in Colorado, Nebraska, Louisiana, and Pennsylvania was positively correlated with daytime temperatures averaged from April–October [9].

In contrast to temperature, the relationship between WNv and precipitation is less straightforward. High precipitation is generally believed to heighten subsequent WNv risk by creating pools of standing water suitable for mosquito oviposition. A study in Rhode Island found that arbovirus prevalence increased in years with higher than average precipitation [10]. However, other studies have found that rainfall has weak correlations with temporal patterns of mosquito abundance [4], [11] and spatial patterns of human WNv cases [9]. A national study found inconsistent correlations between spatial patterns of precipitation and WNv incidence [12]. Both the strength and the direction (positive or negative) of the correlations varied with the geographic area and the time period examined. The degree to which rainfall leads to increased breeding habitats for mosquitoes depends on a variety of local factors, including topography, soils, vegetation, and the particular mosquito species that are present. Alternately, periods of low rainfall may reduce populations of mosquito competitors and predators, leading to larger mosquito populations and higher rates of amplification and transmission the year following the drought [13].

Land cover and land use have also been shown to influence mosquito populations and the risk of WNv transmission. A study of the spatial patterns of five mosquito species implicated as WNv vectors in Connecticut documented habitat relationships with several land cover variables, including forest cover, open water, and distance to estuaries or marshes [14]. A study of the 2002 WNv outbreak in the greater Chicago area found that clusters of human WNv cases were associated with areas of high vegetation cover along with human demographics, distance to WNv positive dead bird specimens, age of housing, landform, and the level of mosquito control [15]. Studies of WNv serostatus in bird communities across the state of Georgia [7] and of WNv incidence during the 2002 WNv outbreak in Chicago and Detroit [16] both found that suburban areas with intermediate levels of population density, housing density, and vegetation cover had higher WNv incidence than more rural and more urbanized areas. In contrast, a study of WNv in Iowa found that WNv incidence was highest in rural areas [17].

We focused our environmental analysis on the 2003 WNv outbreak because of the large number of cases that occurred in our study area during this year (4,490), and because it allowed us to examine environmental relationships prior to the intensification of mosquito control efforts across the region and the development of immunity in both human and avian populations. Thus, patterns of WNv incidence in 2003 should exhibit a stronger association with underlying environmental drivers than in subsequent years. Our overarching hypothesis is that spatial patterns of WNv in the northern Great Plains are determined by environmental drivers, and that these drivers have maintained a relatively stable zone of elevated WNv risk in the northern Great Plains. We tested this hypothesis by applying cluster detection methods to the 2003 WNv outbreak and examining the patterns of WNv cases in subsequent years to see if they fell within the 2003 cluster. We also tested the following set of working hypotheses about the specific environmental drivers of the 2003 WNv outreak: (1) WNv cases are related to weather patterns in 2002, reflecting the influence of prior-year drought on mosquito predators [13]; (2) WNv cases are related to weather patterns in 2003, reflecting the influences of precipitation on breeding habitats and the effects of temperature on mosquito development and the extrinsic incubation period of the virus; (3) WNv cases are related to 30-year climate normals, reflecting the long-term influences of climate on geographic distributions of vector and host species; and (4) WNv cases are related to land cover and land use patterns that affect the availability of mosquito breeding sites, including human population density, irrigated croplands, and wetlands.

## Methods

### Data Sources

**Figure 1:**
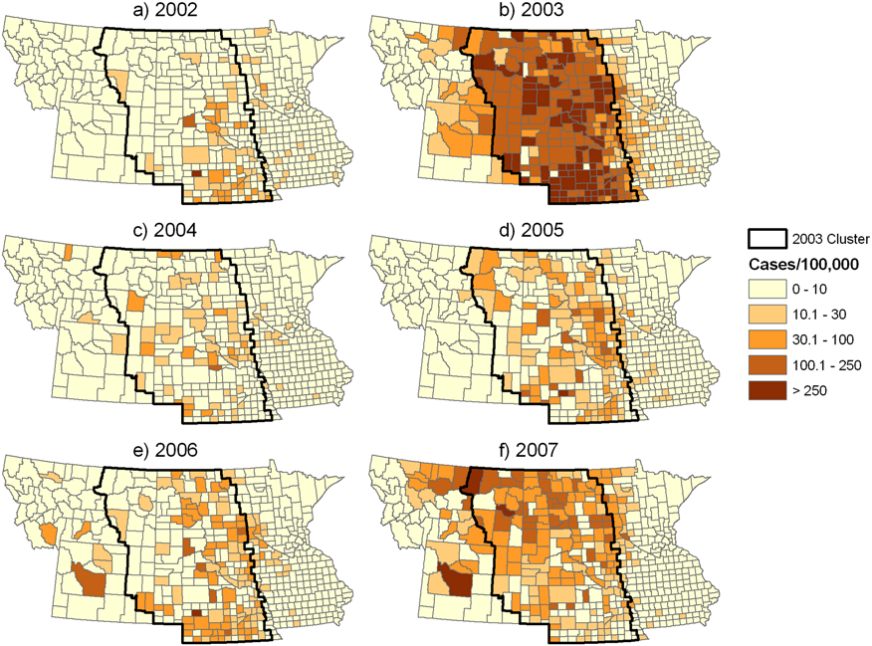
Incidence of WNv expressed as the number of annual cases per 100,000 people. The boundaries of the 2003 cluster were determined using a spatial scan statistic.

WNv cases were obtained from the USGS disease map archive (http://diseasemaps.usgs), which compiles data from the CDC ArboNET surveillance program (Figure 1). The response variable was the total number of reported cases of WNv per county in 2003, including both WNv fever and meningitis/encephalitis. Projected 2003 population data for each county were obtained from the U.S. Census Bureau.

**Figure 2:**
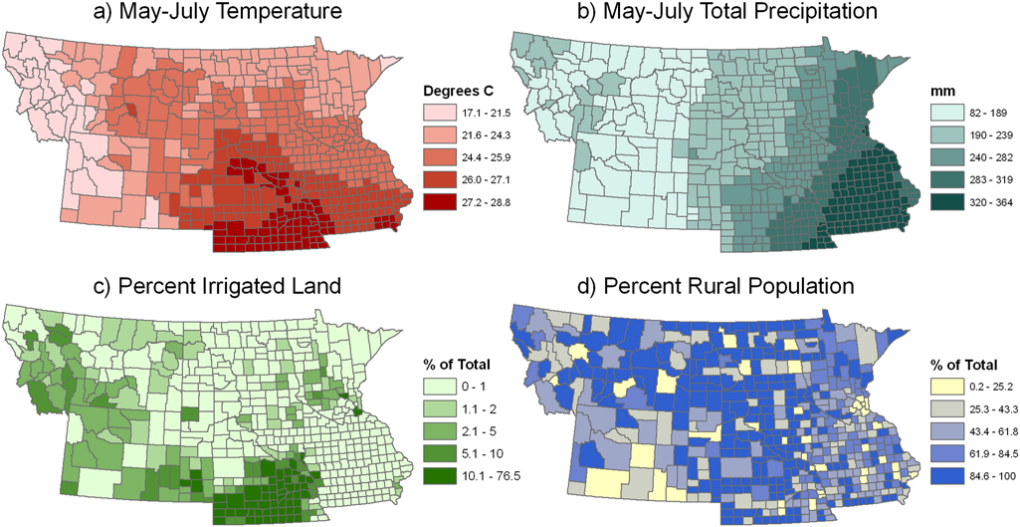
Environmental variables used in the final model of 2003 WNv incidence. a) mean May–July temperature from 1971–2000, b) total May–July precipitation from 1971–2000, c) percent area of irrigated cropland, and d) percent of the population living in rural areas.

Weather variables for 2002–2003 were computed from monthly PRISM weather datasets that characterized mean maximum temperature and precipitation (http://prism.oregonstate.edu). Long-term climate normals were also computed for each month from PRISM climate data that summarized mean maximum temperature and precipitation over the period 1971–2000. The PRISM datasets are gridded weather and climate maps that are generated using a knowledge-based system to interpolate point measurements of temperature and precipitation from weather stations [18], [19]. The gridded PRISM datasets were overlaid with county boundaries to compute mean temperature and precipitation values for each county (e.g., Figure 2a–b).

The percentage of each county covered by wetlands (including both herbaceous and forested wetlands) was computed using the 2001 National Land Cover Dataset [20]. The percentage of each county covered by irrigated cropland was computed using data from the 2002 Census of Agriculture (Figure 2c). The distribution of percent irrigated cropland had a strong positive skew and was square-root transformed prior to analysis. The percentage of the human population in each county living in rural areas was computed using data from the 2000 Census of Population (Figure 2d). The U.S. Census Bureau defines rural areas as being outside of densely populated core areas that have a population of at least 2,500.

### Cluster Analysis

The spatial scan statistic [21] was used to identify clusters of high WNv incidence within the study area. This method uses a variable-sized window to test for possible clusters with varying disease rates. We used an elliptical cluster shape to identify elongated as well as circular clusters. The center of the window was positioned on the centroid of each county, and a range of window sizes and shapes were examined. Neighboring counties were considered to fall within the cluster if their centroids were inside the scanning window. For each window, a likelihood statistic was calculated under the Poisson assumption.
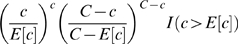



Where *C* was the total number of cases across the entire study area, *c* was the number of cases inside the window, *E*[*c*] was the expected number of cases inside the window under the null hypothesis that the expected number of cases in each area was proportional to population size, and *I* was an indicator function equal to one when the number of cases within the window exceeded the expected value and zero otherwise. The likelihood was maximized across possible cluster locations, sizes, and shapes to identify the most likely disease cluster. A test of statistical significance was carried out using a Monte Carlo simulation of 9,999 random datasets generated under the null hypothesis. Cluster detection was carried out using the SaTScan™ software package [22].

### Statistical Modeling

A statistical model of 2003 WNv incidence was developed using a two-step process. The first step was a comparison of alternative models based on either weather or climate variables. Each model was a second-order trend surface of temperature and precipitation
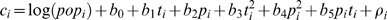
where *c* was the number of cases in county *i*, *pop* was the population size, *t* was the temperature variable, *p* was the precipitation variable, *b* were parameters, and *ρ* was a spatially autocorrelated random effect. The trend surface allowed for temperature and precipitation to have curvilinear or unimodal relationships with WNv incidence, and also captured possible interactions between temperature and precipitation.

Five models were compared: one model based on the 1971–2000 climate normals, two models based on 2002 and 2003 weather, and two models based on 2002 and 2003 weather anomalies. The weather anomalies were computed as the difference between temperature and precipitation in 2002 and 2003 and the 1971–2000 climate normals. In all models, temperature variables were computed as mean May–July temperature and the precipitation variables were computed as the total May–July precipitation. These months were chosen to encompass the main period of WNv amplification in the northern Great Plains, following the emergence of mosquitoes in May and prior to the peak period of human transmission in late July and August.

A hierarchical Bayesian approach was used to model WNv incidence as a function of the alternative sets of weather and climate variables. Spatial random effects based on a conditional autoregressive (CAR) process were incorporated into the models to correct for biases in estimates of the coefficients and their standard errors that occur as a result of spatial autocorrelation [23]–[25]. Noninformative prior distributions were used for all model parameters. Models were fitted via Markov Chain Monte Carlo (MCMC) simulation using WinBUGS software [26]. To improve model convergence and ease of parameter interpretation, all independent variables were standardized by subtracting their means and dividing by their standard deviations. The deviance information criterion (DIC) was used as a metric to compare the candidate models [27]. DIC is computed as the expected deviance over the posterior distribution of the model parameters plus the effective number of parameters in the model. The model with the lowest DIC was selected as having the best fit.

In the second step, a final model was created by adding the three land cover and land use variables (percent irrigated land, percent rural population, and percent wetlands) to the best-fitting temperature and precipitation based model. Non-significant trend surface components were eliminated from the final model. Model fitting and selection was carried out as described in the previous step.

To aid in visualizing non-linear relationships between WNv incidence and environmental variables, we created contour plots using local regression models [28]. This method uses polynomial functions to create a smoothed, nonparametric representation of the response surface. The standardized incidence rate for each county was computed as
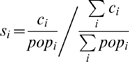
where the quantity in the numerator is the incidence rate for county *i*, and the quantity in the denominator is the incidence rate for the entire study area. The dependent variable for the local regression models was then computed as log (*s_i_*+1).

## Results

### Clustering

Cluster analysis of the 2003 WNv epidemic identified a single elliptical cluster encompassing most of North Dakota, South Dakota, and Nebraska along with portions of eastern Montana and Wyoming. The relative risk and 95% confidence interval for populations inside the 2003 WNv cluster compared with those outside the cluster was 7.3 (5.8–9.1) in 2002, 24.2 (22.4–26.1) in 2003, 7.4 (5.5–9.8) in 2004, 13.4 (11.3, 16.0) in 2005, 9.5 (8.2–11.1) in 2006, and 7.6 (6.8–8.5) in 2007. These statistics, combined with overlay maps of the 2003 WNv cluster boundary with incidence rates from 2004–2007 (Figure 1), indicated that the broad zone of elevated WNv morbidity in the northern Great Plains remained relatively stable over this time period. However, within the boundary of the 2003 WNv cluster, different areas emerged with high incidence rates in different years. Several counties in Montana and Wyoming located outside of the 2003 WNv cluster had intermediate incidence rates in 2003, low incidence rates in 2004 and 2005, and relatively high incidence rates in 2006 and 2007.

### Statistical Modeling

**Figure 3:**
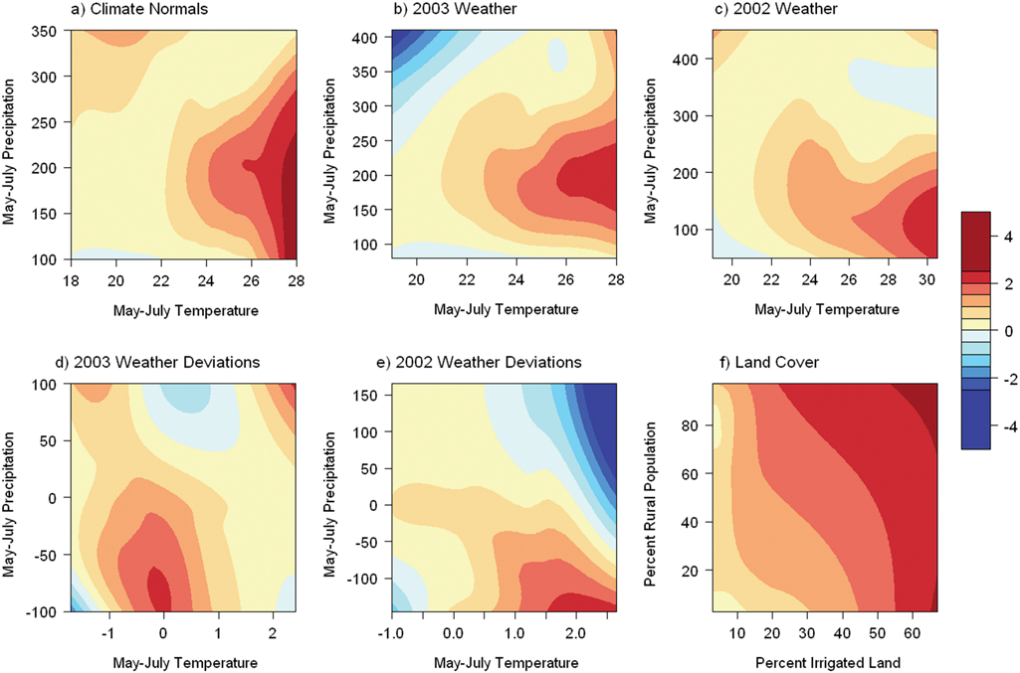
Logarithm of the standardized incidence rate as a function of environmental variables. The smoothed response surfaces were generated using local regression models.

The model based on 1971–2000 climate normals had the best fit, as indicated by the lowest DIC value (Table 1). In the climate-based model, WNv incidence had a positive linear relationship with temperature and a unimodal relationship with precipitation, whereas the relationships with the quadratic temperature term and the interaction term were not statistically significant (Figure 3a). The models based on 2003 weather variables and weather anomalies had weaker fits than the model based on climate, as indicate by higher DIC values. The 2002 models had the weakest fits. In both the 2003 weather model (Figure 3b) and the 2002 weather model (Figure 3c), WNv incidence had a positive linear relationship with temperature and a unimodal relationship with precipitation that was similar to the responses in the climate model. In the 2003 weather anomaly model, WNv incidence had a negative relationship with the quadratic temperature term and was highest where temperature deviations were close to zero (Figure 3d). In the 2002 weather anomaly model, WNv incidence had a negative relationship with the quadratic precipitation term and was highest where 2002 precipitation was below normal (Figure 3e).

**Table 1:** Trend surface models of 2003 WNv cases as a function of temperature and precipitation.

Variable	Model				
	1971–2000 Climate	2003 Weather	2002 Weather	2003 Weather Anomalies	2002 Weather Anomalies
*Intercept*	−7.949*	−8.096*	−8.059*	−8.082*	−8.486*
*t*	1.083*	0.581*	0.934*	−0.255	0.192
*p*	−0.851*	0.153	0.007	0.119	−0.094
*t* ^2^	−0.039	−0.012	−0.010	−0.241*	−0.201
*p* ^2^	−0.553*	−0.273*	−0.259*	−0.039	−0.252*
*pt*	0.291	0.026	0.130	0.054	−0.432
*σ_ρ_*	1.537*	1.610*	1.557*	1.640*	1.669*
DIC	2014.0	2019.4	2020.6	2018.1	2023.0

*t* = mean May–July temperature, *p* = total May–July precipitation, *σ_ρ_* = standard deviation of the spatial random effect.

* statistically significant at the *p* = 0.05 level.

Eliminating non-significant climate variables and incorporating land cover and land use variables substantially improved the fit of the final model when compared to the climate models, as indicated by a substantial reduction in DIC (Table 2). The final model indicated that 2003 WNv incidence had positive linear relationships with irrigated cropland and percent rural population (Figure 3f) in addition to a positive relationship with temperature and a unimodal relationship with precipitation. The effect of wetlands on WNv cases was relatively weak and was not statistically significant. Based on the second-order polynomial relationship with precipitation, the optimum May–July precipitation was approximately 200 mm. Counties with this precipitation were predicted to have the highest levels of WNv incidence, with decreasing incidence at both lower and higher precipitation.

**Table 2:** Final model of 2003 WNv cases as a function of climate and land cover/land use.

Variable	Parameter	95% Bayesian credible interval	
		5%	95%
*Intercept*	−7.917	−8.132	−7.690
*t*	0.877	0.608	1.149
*p*	−0.695	−1.212	−0.147
*p* ^2^	−0.409	−0.629	−0.204
*irrigated*	0.337	0.194	0.483
*prural*	0.206	0.128	0.284
*wetland*	0.002	−0.158	0.162
*σ_ρ_*	0.504	0.400	0.623
DIC	2007.4		

*t* = mean May–July temperature from 1971–2000, *p* = total May–July precipitation from 1971–2000, *irrigated* = percent area of irrigated cropland, *prural* = percent of the population living in rural areas, *wetland* = percent area of wetlands, *σ_ρ_* = standard deviation of the spatial random effect.

## Discussion

Analysis of the 2003 WNv epidemic in the northern Great Plains using the spatial scan statistic identified a single, large cluster of high WNv incidence. In the statistical models, temperature, precipitation, percent irrigated land, and percent rural population were all correlated with this pattern of regional clustering. In subsequent years, the majority of WNv cases occurred within the boundaries of the 2003 WNv cluster, although WNv incidence was much lower after 2003. This decrease in WNv cases following the initial epidemic year likely reflects multiple factors including increases in immunity both in bird and human populations, increased efforts at mosquito control by municipalities, and enhanced public awareness and preventative measures by individuals. However, WNv incidence in the northern Great Plains has remained high compared to other parts of the United States [2], suggesting that the biogeographic characteristics of this region favor the maintenance and continued transmission of WNv.

We interpret the results of our environmental models as evidence that the cluster of high WNv incidence during the 2003 epidemic, as well as the sustained high incidence of WNv in subsequent years, is linked to the geographic distribution of a key vector species *Culex tarsalis*. This species is known to be a particularly efficient vector of WNv [29], and has been implicated as the primary vector of WNv in the northern Great Plains [2], [30], [31]. We are not able to prove this association with *Culex tarsalis* in our analysis of human cases, but the environmental relationships we observed offered several lines of evidence in support of this hypothesis. The pattern of the 2003 WNv outbreak had stronger relationships with long-term climate normals than with weather or weather anomalies in either 2002 or 2003. Although vector and host abundances can vary with annual weather fluctuations, the geographic range boundaries of vector and host species are typically more stable and are linked to longer-term climate patterns [32]. Therefore, it is more likely that the spatial cluster of WNv cases in 2003 reflects the geographic distribution of one or more vector or host species than a short-term, weather-driven increase in their populations. The stability of the WNv cluster in subsequent years further supports this interpretation.

Most other studies have examined monotonic relationships between precipitation and WNv incidence based on an underlying assumption that either high rainfall will provide favorable breeding sites [10] or that drought may affect the spatial patterns of vector and host populations [13]. In contrast, we found a strong unimodal relationship between precipitation and WNv incidence. This relationship is consistent with the predictions of ecological niche theory, which posits that different species achieve their peak abundance at different points along environmental gradients [33]. The results of our analysis suggest that a total May–July precipitation of approximately 200 mm reflects optimal conditions for WNv amplification and transmission in the northern Great Plains. As precipitation increases to the east, the importance of *Culex tarsalis* in mosquito communities decreases and it is replaced by less efficient vectors such as *Aedes vexans* and *Culex pipens* [17]. As precipitation decreases to extremely low levels, breeding habitat for all mosquitoes decreases, also reducing WNv transmission risk. The positive relationship with temperature likely reflects influences on mosquito development and extrinsic incubation rates [3].

The relationships between WNv incidence and rural land use highlight key differences between the dynamics of WNv in the northern Great Plains versus other parts of the United States. Research in the eastern and midwestern United States has primarily focused on *Culex pipens* as a vector species, and has reported higher WNv incidence and seroprevalence in urban and suburban habitats than in more rural areas [7], [16], [34]. In contrast, our research and another study in Iowa [17], found that WNv incidence was highest in rural areas. We further found that WNv incidence increased with the percent of irrigated land in rural areas. The association of *Culex tarsalis* with rural areas and irrigated land has been documented both in the Great Plains [35] and California [36] further supporting the hypothesis that the geographic distribution of *Culex tarsalis* is a major determinant of the regional patterns of WNv incidence.

As with all spatial ecological and epidemiological studies, the interpretation of our results is contingent upon the temporal and spatial scales of the analysis [37], [38]. We focused our analysis on the drivers of spatial patterns of WNv cases within a single epidemic year, and we examined these relationships at a fairly coarse spatial resolution (counties) and across a large spatial extent (7 states encompassing 1,578,000 km^2^). Although we found that weather variables in 2002 and 2003 were relatively weak predictors of the spatial distribution of WNv cases in 2003, these variables may still be important in explaining year-to-year variability in the number and spatial distribution of WNv cases from 2004-present. Analyses focused at smaller spatial resolutions also have the potential to reveal environmental relationships that are not apparent at the county level [37]. In particular, relationships with wetlands and other sources of local breeding habitat may be more evident at finer spatial resolutions [39]. Finally, the strength and even the direction of some environmental relationships may vary spatially across large study areas, and analyses that focus on subregions or techniques such as geographically weighted regression (GWR) may provide novel insights [25], [40].

Based on our analysis of the 2003 WNv epidemic in the northern Great Plains, we found multiple lines of evidence to support the hypothesis that the geographic distribution of *Culex tarsalis* is at least partially responsible for the continued high levels of WNv incidence in this region. However, the mere presence of a vector species is not sufficient to sustain transmission of a zoonotic disease. Studies have documented a wide range of variability in avian host competence for WNv [41], and it is probable that spatial variability in the abundance and community composition of avian hosts has an effect on the potential for amplification of WNv and the consequent patterns of human cases in the northern Great Plains. At this point, much of our understanding of the complex zoonotic cycles that sustain WNv is derived from studies conducted in the eastern and midwestern United States where the primary WNv vectors, human demographics, and environmental patterns are fundamentally different from the northern Great Plains. The existence of a sustained cluster of elevated WNv incidence encompassing North Dakota, South Dakota, and Nebraska along with portions of eastern Montana and Wyoming highlights the need for more epidemiological research in this region to determine the proximal causes of sustained WNv transmission and to enhance strategies for disease prevention.
